# Regulations of Retinal Inflammation: Focusing on Müller Glia

**DOI:** 10.3389/fcell.2022.898652

**Published:** 2022-04-27

**Authors:** Yingying Chen, Qinghong Xia, Yue Zeng, Yun Zhang, Meixia Zhang

**Affiliations:** ^1^ Department of Ophthalmology, Sichuan University West China Hospital, Sichuan University, Chengdu, China; ^2^ Research Laboratory of Macular Disease, West China Hospital, Sichuan University, Chengdu, China; ^3^ Operating Room of Anesthesia Surgery Center, West China Hospital, Sichuan University, Chengdu, China; ^4^ West China School of Nursing, Sichuan University, Chengdu, China

**Keywords:** retinal inflammation, Müller glia, cytokines, miRNA, regeneration

## Abstract

Retinal inflammation underlies multiple prevalent retinal diseases. While microglia are one of the most studied cell types regarding retinal inflammation, growing evidence shows that Müller glia play critical roles in the regulation of retinal inflammation. Müller glia express various receptors for cytokines and release cytokines to regulate inflammation. Müller glia are part of the blood-retinal barrier and interact with microglia in the inflammatory responses. The unique metabolic features of Müller glia in the retina makes them vital for retinal homeostasis maintenance, regulating retinal inflammation by lipid metabolism, purine metabolism, iron metabolism, trophic factors, and antioxidants. miRNAs in Müller glia regulate inflammatory responses via different mechanisms and potentially regulate retinal regeneration. Novel therapies are explored targeting Müller glia for inflammatory retinal diseases treatment. Here we review new findings regarding the roles of Müller glia in retinal inflammation and discuss the related novel therapies for retinal diseases.

## Introduction

### Retinal Inflammation

The vertebrate retinas are evolutionarily conventional and highly organized for normal function (Hoon et al., 2014). The vasculature in the human retina begins to develop since the first month of gestation (Lutty and McLeod, 2018) and ends up being a laminar meshwork of capillaries that permeates the inner neural retina (Selvam et al., 2018). The outer neural retina is supported by the choroidal vasculature instead (Selvam et al., 2018). Both inner and outer retina is blocked from direct contact with vasculature by the retinal-blood barrier (BRB). BRB contributes to the immune privilege of the retina and suppresses retinal inflammatory responses with the immune suppressive microenvironment (Chen et al., 2019). Retinal inflammation occurs when the immune system is activated by intrinsic or extrinsic antigens, manifesting as retinitis or uveitis with impaired visual acuity, dyschromatopsia, relative afferent pupillary defects and visual field abnormalities (Abdelhakim and Rasool, 2018; Wildner and Diedrichs-Mohring, 2019). Retinal inflammation can also be induced by chronic para-inflammation, which contributes to the initiation and progression of prevalent retinal diseases including but not limited to diabetic retinopathy (DR), age-related macular degeneration (AMD) and glaucoma (Xu et al., 2009). The macular edema, which is resulted from breakdown of the BRB, a characteristic of inflammation in the neural retina, is one of the commonest causes of vision loss in DR (Daruich et al., 2018). The consistent age-related para-inflammation in the retina can lead to inflammatory responses, contributing to the pathogenesis of AMD (Xu et al., 2009). Drusen in AMD retina are signs of inflammation as they are composed of multiple inflammation-related components including lipids and proteins that are involved in the inflammatory sequelae and complements (Johnson et al., 2001; Anderson et al., 2002). The size of drusen is used to evaluate the severity of AMD, indicating its significance (Ferris et al., 2005). Glaucoma is characterized by deaths of the ganglion cells, which inevitably induces inflammation in the retina (Xu et al., 2009). Inflammation in the anterior chamber can also be observed in the acute phase of glaucoma, including ciliary injection and cloudy aqueous humor. The control of intraocular pressure is not always effective for glaucoma treatment, indicating the significance of other manipulations, such as inflammation regulation (Xu et al., 2009).

### Müller Glia

Müller glia (MG) are the most abundant glial cells in the retina, with processes spanning the entire thickness of the neural retina, contributing to the formation of the inner limiting membrane (ILM) and the outer limiting membrane (OLM) (Reichenbach and Bringmann, 2020) and maintaining the integrity of the BRB (Kugler et al., 2021). The unique spanning structure makes MG interact with every kind of retinal neuron, contributing to the water and electrolytes balance, trophic support of retinal neurons, and neurotransmitter recycling, while new functions are being found (Reichenbach and Bringmann, 2013).

MG play critical roles in retinal inflammation. MG maintain normal retinal structure (Byrne et al., 2013), support neuron survival and vascular health in the retina (Shen et al., 2012), while the disrupted retinal structure and vascular dysfunction contribute to the changed immune microenvironment and facilitate the infiltration of immunocytes from the vessel (Forrester et al., 2020). MG can undergo gliosis within inflamed retina, which can be both beneficial and detrimental. Gliotic MG secrete neuroprotective factors and upregulate the expression of nitric oxide (NO) synthase that enhance NO production and promote cell survival via dilating blood vessels and close N-methyl-d-aspartate (NMDA) receptor channels (Bringmann et al., 2009). Hypertrophied fibers in gliotic MG contribute to the gliotic scar in the retina that isolates diseased area (Bringmann et al., 2006). On the other side, the gliotic scar disrupts the normal retinal structure and increases the risk of retinal detachment (Bringmann et al., 2006). The neuroprotective factors can be detrimental as well, such as VEGF. Gliotic MG express less functional proteins such as glutamine synthetase (GS) and inwardly rectifying potassium (Kir) channels (Bringmann et al., 2009). MG dysfunction also compromise BRB (Bringmann et al., 2009). Both contribute to the disturbed the fluid homeostasis and cause macular edema and lead to vision loss.

The functional and structural changes in MG lead to changes in the intrinsic and extrinsic cells that interact with MG. For example, gliotic MG in DR, when suffered from altered expression of Kir channel, absorb less of the extracellular potassium released by retinal neurons and contribute to their altered cellular excitability. Decreased expression of GS causes increased glutamate level, which adds to the toxicity of the neurotransmitters to the neurons. Activated MG produce pro-inflammatory factors, some of which recruit extrinsic immunocytes to the retina, where the BRB is compromised because of increased cellular permeability and altered ECM. The recruited immunocytes secrete inflammatory factors that regulate the responses of activated MG (Coughlin et al., 2017). MG in the injured retina interact with microglia, the widely accepted player in inflammation in the central nervous system (Saijo and Glass, 2011), to regulate the injury-induced retinal inflammation (Wang and Wong, 2014).

MG participate in the recognition and phagocytosis of pathogens and antigen-presenting during infection, constituting the first line of defense in the retina and induce inflammatory responses (Kumar et al., 2013; Singh et al., 2014; Bejarano-Escobar et al., 2017; Lorenz et al., 2021; Schmalen et al., 2021).

MG interplay with retinal inflammatory responses of the retina via complicated mechanisms. Here, we tried to talk about the roles MG played in different facets of the retinal inflammation and the possible treatments aiming at the MG function rescue.

## Cytokines and the MG

Cytokines are important inflammatory mediators, playing complicated roles in the pathogenesis of retinal diseases. For example, the levels of IL-1β, IL-6, IL-8, and tumor necrosis factor (TNF)-α are higher in eyes with DR. Interestingly, inflammatory cytokine and neurotrophic factor levels are higher in eyes with non-proliferative diabetic retinopathy (NPDR) than in eyes with active PDR, although the authors stated that serum/plasma proteins might have leaked into the vitreous and diluted the relative amount of NTs and cytokines in PDR patients. (Boss et al., 2017). In the normal retina, high expression of fibroblast growth factor basic (FGFb, FGF2), granulocyte-macrophage colony-stimulating factor (GM-CSF), interferon-gamma-induced protein (IP)-10 (also known as CXCL10), and granulocyte colony-stimulating factor (G-CSF) have been observed, while the levels of GM-CSF and G-CSF significantly increase in the PVR specimens (Eastlake et al., 2016). MG are important source of inflammatory cytokines, capable of secretion of more than all the cytokines above. MG secretomes show profound and distinct response profiles after stimulation with pro-inflammatory cytokines like IFN-γ or TNF-α, or with growth factors like TGFβ or VEGF, thus confirming their central role of cellular communication and chronic inflammation in the retina (Schmalen et al., 2021). In this section, we’ll focus on the interaction between the MG and the interleukin families, the chemokine families, and the growth/neurotrophic factors.

### IL-1 Family and the MG

Interleukins are important cytokines in retinal inflammation. The IL-1 family is a central mediator of innate immunity and inflammation, with 11 cytokines and 10 receptors (Wooff et al., 2019). One of the most studied cytokines is the IL-1β. MG can express IL-1β in human MG *in vitro* (Giblin et al., 2021). Both IL-1R and IL-1R1-associated signal-transduction genes can be found in MG from NMDA-damaged mouse retinas (Todd et al., 2019). Responses to IL-1β also suggest the present of IL-1R in MG. Stimulated by IL-1β, MG produces more chemokines (Ccl2, Cxcl1, and Cxcl10), which are involved in retinal degeneration (Natoli et al., 2017). IL-1β induces expression of IL-6 and IL-8 in MG (Liu et al., 2014; Liu et al., 2015), which are positively related to many retinal diseases, including but not limited to DR, AMD, central retinal vein occlusion (CRVO) (Ghasemi, 2018). IL-1β has been reported to induce both TNF-α and IL-1β expression in MG, both contribute to MG inflammation (Giblin et al., 2021). IL-18 is another member of the IL-1 family expressed by MG (Zhang et al., 2022), well known for its pro-inflammation functions (Kaplanski, 2018), and is found to participate in age-related neurodegenerative diseases with IL-1β (Brahadeeswaran et al., 2022). Being one of the IL-1 family, IL-33 is also shown to be mainly expressed by MG in the neural retina, which is elevated in AMD patients and regulates mononuclear phagocyte recruitment to the photoreceptor layer by increasing inflammatory chemokine and cytokine expression (Xi et al., 2016). IL-33-deprived mice show enhanced retinal degeneration and gliosis following retinal detachment (RD), which is related to sustained subretinal inflammation from infiltrating macrophages (Augustine et al., 2019). Local administration of recombinant IL-33 was reported to inhibit murine choroidal neovascularization (CNV) formation, indicating its modulative function in neovascular AMD (Theodoropoulou et al., 2017).

### IL-6 Family and the MG

In the retina, IL-6 shows pleiotropic functions, such as neural retina protection and retinal barrier disruption (Coughlin et al., 2019). IL-6 functions via binding the membrane-bound IL-6R (mIL-6R, classical IL-6 signaling), or via the soluble IL-6R (sIL-6R, IL-6 trans-signaling) in mIL-6R (-) cells that express the cytokine receptor gp130 (Neurath and Finotto, 2011). The protective effects of IL-6 on MG vary between classical and trans-signaling pathways, with different dependencies on VEGF-A signaling (Coughlin et al., 2019). Expressing mIL-6R/gp130 and releasing the sIL-6R/soluble gp130, MG are active both in the IL-6 signaling (Coughlin et al., 2019). Ablation of MG in mice induces profound changes in the expression of IL-6/gp130 cytokines and downstream signaling, indicating an essential role of MG in this signaling (Coorey et al., 2015). Other members of the IL-6 family also work with MG to show their neuroprotective effect. CNTF, a member of the IL-6 family, is involved in the photostasis, development, differentiation, and survival of retinal neurons (Wen et al., 2012). Only with the activation of the gp130 in MG, can CNTF-mediated protection of photoreceptors be observed (Rhee et al., 2013). In response to CNTF treatment, the JAK/STAT3 signaling pathway, which plays a crucial role in the mediation of inflammatory responses, is activated in MG with reduced outer retinal neovascularization (Bucher et al., 2020). CNTF protects the retinal neurons from toxic insult but also suppresses damage-induced proliferation of MG in the retina of zebrafish (Fischer et al., 2004). Leukemia inhibitory factor (LIF) is another pleiotropic member of the IL-6 family (Nicola and Babon, 2015). The expression of LIF and its receptors are confirmed in MG in mice (Pannicke et al., 2018). Together with CNTF, LIF shows neuroprotective and axon growth-promoting effects following inflammatory stimulation on mature retinal ganglion cells in mice (Leibinger et al., 2009), which also requires the activation of gp130 and signaling via the JAK/STAT3 pathway (Kirsch et al., 2010). Lif expression in mouse MG and retina is inducible by TNF-α and is neuroprotective during photoreceptor degeneration through the p38 MAPK pathway (Agca et al., 2013). Oncostatin M (OSM), another member of the IL-6 family of cytokines, protects photoreceptors in an MG-dependent manner (Xia et al., 2011).

### Other ILs and the MG

Although there’s no direct evidence for IL-4 receptor expression in MG, pro-inflammatory proteins like IFNγ, S100A9, S100A7, CXCL10, and Lysozyme C (LYZ) increase upon stimulation of MIO-M1 cells with IL-4, indicating that IL-4 has a pro-inflammatory influence in MG (Schmalen et al., 2021). IL-17RA, as well as IL-17A, expresses in MG (Qiu et al., 2016). Both can be induced by diabetes, which enhances the inflammatory Act1/TRAF6/IKK/NF-κB signaling pathway activation (Qiu et al., 2016), and contributes to retinal ganglion cells (RGCs) apoptosis (Qiu et al., 2021). Following antigen stimulation or activation by cytokines such as IL-1 and TNF-α, G-CSF and GM-CSF are produced and generated by different types of cells and promote the production of granulocytes or antigen-presenting cells (APC) (Mehta et al., 2015). Both cytokines can be expressed by MG (Eastlake et al., 2016). In the retina, G-CSF exhibits neuroprotective effect, especially on the optic never injury (Liu et al., 2020; Huang et al., 2021), and vasculopathy (Kojima et al., 2011). Sharing a common β-chain with IL-3 and IL-5, *in vitro* studies suggest that GM-CSF reduces oxidative stress in retinal pigment epithelium (RPE) (Bascuas et al., 2021), promotes regeneration of retinal ganglion cells (Legacy et al., 2013). GM-CSF also protects rat photoreceptors from death *in vivo* (Schallenberg et al., 2012).

### Chemokines and the MG

The chemokine family consists of approximately 50 proteins that are divided into 4 subgroups: CXC, CC, C, and the CX3C chemokines, depending on their structure (Bruserud and Kittang, 2010). MG are the early source of the chemokine Ccl2 (also known as monocyte chemoattractant protein (MCP)-1) after light damage to rat retina, which facilitates targeting of monocytes to sites of injury (Rutar et al., 2011). The suppression of Ccl2 in MG, mediated by siRNA, attenuates detrimental inflammatory responses by reducing microglial recruitment and reduces photoreceptor death following rat retinal degeneration (Rutar et al., 2012). MG also express Ccl7, Cxcl1, and Cxcl10 after light damage, which recruit neutrophils, T cells, monocytes/microglia and participate in the inflammatory responses (Rutar et al., 2015). CX3CL1 secretion from the MG increases after toxic insult with systemic N-methyl-N-nitrosourea (MNU) administration in rats, while the expression of its receptor CX3CR1 increased in microglia (Zhang S. et al., 2018), which is another evidence showing the close interaction between the MG and microglia regarding inflammatory responses. CXCL10 level was found to be elevated in patients with all stages of AMD (Mo et al., 2010). Vitreous CXCL10 level in PDR patients is higher than the normal people and is involved in the termination of angiogenesis and promotion of fibrosis (Nawaz et al., 2013). However, a mouse study reported that the intravitreal injections of recombinant CxCl10 significantly reduced outer retinal neovascularization (Bucher et al., 2020). Taken together, the modulation of the CXCL10 level is a potential target for angiostasis control in the retina. CXCL16 can target MG and induce upregulation of the p65 subunit of NF-κB and phospho-ERK1/2 and synthesis and secretion of VEGF (Abu El-Asrar et al., 2020). Other cytokines can share the receptors with the chemokines. The cytokine macrophage migration inhibitory factor (MIF) plays a critical role in inflammatory diseases and atherogenesis via the receptors CD17, CXCR2, and CXCR4 (Bernhagen et al., 2007; Su et al., 2017). MG are both source and target of MIF, participating in the regulation of retinal inflammatory responses (Abu El-Asrar et al., 2019).

### The TNF Superfamily and the MG

The TNF superfamily, composed of 19 ligands and 29 receptors that are all pro-inflammatory, plays various roles in the body (Aggarwal et al., 2012). TNF-α and its receptors are among the most studied members of this family, especially in retinal inflammation (Jiang et al., 2021). TNF-α primarily exists as a transmembrane protein, which binds and activates TNFR1 and TNFR2, while the TNF-α converting enzyme (also known as ADAM17, which also cleaves TNFRs to produce soluble TNFRs) can cleave the transmembrane TNF-α into a soluble form (sTNF-α), which activates primarily TNFR1 (Jiang et al., 2021). TNF-α expression has been observed in MG and it is highly inducible by stimulations like IL-1β, as discussed above. TNFR1 and TNFR2 expression in MG has also been reported, which can be inhibited and result in decreased NF-κB inflammatory responses (Ji et al., 2022), indicating their critical roles in the inflammatory responses. P38MAPK and JNK signaling pathways are also downstream of the TNFR, involving in important cellular functions including cell survival, proliferation and immune defense, ultimately lead to deaths of retinal neuron and vasculopathy (Jiang et al., 2021). Interestingly, mammals and zebrafish react differently to the same factor. In mice, TNF-α induces immune cell infiltration into the vitreous as well as vasculitis, subsequently inducing the development of fibrosis and epiretinal membranes (Weigelt et al., 2021). In zebrafish, however, TNF-α released by dying retinal neurons induces the resident MG to reprogram and re-enter the cell cycle (Lahne and Hyde, 2017), indicating the possibility of treating retinal inflammation by manipulating the inflammatory responses of MG. CD40 is another member of the TNF family. Intravitreal administration of a cell-penetrating CD40-TRAF2,3 blocking peptide impaired CXCL1 upregulation in endothelial and MG, diminished inflammation and neuronal loss after ischemia/reperfusion, as mentioned above (Portillo et al., 2021). CD40 expression in MG can be increased by diabetes (Dierschke et al., 2020), which amplifies inflammation and induces death of retinal endothelial cells, an event key to the development of capillary degeneration and retinal ischemia (Subauste, 2019).

### Growth/Neurotrophic Factors and the MG

VEGF is perhaps the most studied growth factor in the retina, which will be talked about later in this review together with factors that are related to BRB. The TGFs, primarily the TGF-β, are important growth factors regulating many cellular activities. The TGF-β superfamily has three multifunctional isoforms TGF-β1, TGF-β2, and TGF-β3 (Conedera et al., 2021). TGF-β1 stimulated TGF-β-SNAIL axis induces MG-mesenchymal transition (MET) in the pathogenesis of idiopathic epiretinal membrane in mammals, indicating its roles in retinal fibrosis (Kanda et al., 2019). TGF-β1/2 administration causes increased phosphorylation of SMAD3 and p38MAPK with increased VEGF-A mRNA expression in MG, which has been observed in fibrovascular tissues from patients (Wu D. et al., 2020). TGF-β1 and Notch have been shown to promote MG-mediated retinal gliosis in mice (Fan et al., 2020). TGF-β3 in MG is upregulated after retinal injury and promotes retinal regeneration in zebrafish, while in mice it is the TGF-β1/2 that are upregulated and activate MAPK pathway, causing retinal fibrosis (Conedera et al., 2021). Studying the different patterns of TGF-β signaling between mammals and the regenerative zebrafish can be a target for retinal diseases therapy. Norrin is a secreted signaling molecule with structural and functional characteristics of an autocrine and/or paracrine acting growth factor. In the eye, Norrin is constitutively expressed in MG (Ohlmann and Tamm, 2012). Neuroprotective effects of Norrin similarly involve activation of Wnt/β-catenin signaling and the subsequent induction of neuroprotective growth factor synthesis in MG, such as that of FGF2 or ciliary neurotrophic factor (CNTF) (Seitz et al., 2010).

Levels of vitreous neurotrophic factors of DR patients were significantly higher than those of nondiabetic controls (nerve growth factor (NGF), brain-derived neurotrophic factor (BDNF), neurotrophin-3 (NT-3), NT-4, CNTF, and glial cell-derived neurotrophic factor (GDNF) (Boss et al., 2017). The basal production of NGF is low but can be enormously upregulated in MG during an inflammatory response or tissue damage (Telegina et al., 2019). The NGF receptor, p75 neurotrophin receptor (p75^NTR^) is a member of the TNFR superfamily, involved in nuclear factor κB (NFκB) activation and production of proinflammatory mediators (Mysona et al., 2013). The receptor is expressed predominantly by MG. The inhibition of the receptor prevents retinal inflammation and blood-retina barrier breakdown in mice and rats induced by diabetes or NGF precursor (Mysona et al., 2013). GDNF, like LIF, is a growth factor that is secreted by MG and regulated by microglia under inflammatory responses (Wang et al., 2011). GDNF has neuroprotective effects on photoreceptors and RGCs (Fudalej et al., 2021). Therapies aiming to increase GDNF expression in the retina were reported, including recombinant adeno-associated virus expressing GDNF (rAAV-GDNF) injection in rat eyes (Wu et al., 2002), electrotransfer of GDNF-encoding plasmid in the rat ciliary muscle (Touchard et al., 2012), intravitreally administration of GDNF slow-release formulation (Garcia-Caballero et al., 2018), intravitreal injection of genetically engineered stem cells that oversecrete GDNF (Gregory-Evans et al., 2009). The studies showed some promising results, while more investigations are needed for retinal diseases treatment.

Midkine (MDK) and pleiotrophin (PTN) are neurotrophic factors that belong to a family of basic heparin-binding cytokines (Gramage et al., 2014). MDK was found to be upregulated in proliferating MG in zebrafish and chick retina but was decreased in mouse MG after NMDA damage (Campbell et al., 2021). While MDK promotes the migration of inflammatory cells (macrophages and neutrophils) (Muramatsu, 2002), MDK treatment promotes an increase in proliferating MG progenitor cells in damaged mouse retinas and potently decreased dying cells (Campbell et al., 2021). With the inducible expression of MDK-receptors, including ITGB1, PTPRZ, in the MG, microglia, and other retinal cells, the MDK signalling pathway in the retina is dynamically regulated during the development and stress responses of the retina and manipulate multiple cell functions including proliferation, migration (Muramatsu, 2002; Campbell et al., 2021; Qi et al., 2021). While pigment epithelium-derived factor (PEDF) locates in the interphotoreceptor matrix and is believed to be a neuroprotective factor released by RPE (Polato and Becerra, 2016; Pagan-Mercado and Becerra, 2019), down-regulation of PEDF expression in MG resulted in increases of VEGF and TNF-α secretion, indicating PEDF an endogenous anti-inflammatory factor in MG (Zhang et al., 2006).

Table 1 concluded some typical MG-related cytokines and how they regulate retinal inflammation.

**TABLE 1:** MG-related cytokines and retinal inflammation.

Cytokines (and receptors)	Main functions related to retinal inflammation	Type of studies and references
IL-1β and receptors, IL-8, Il-18	To induce cytokines secretion and promote inflammation	*In vitro* human MG studies Liu et al. (2014), Liu et al. (2015), Natoli et al. (2017), Giblin et al. (2021), Zhang et al. (2022)
		*In vivo* mice study Todd et al. (2019)
IL-33	To recruit macrophages; inhibit CNV	Human retina, *in vivo* murine and *in vitro* rat MG studies Xi et al. (2016), Theodoropoulou et al. (2017), Augustine et al. (2019)
IL-6, CNTF, LIF, OSM	To protect neural retina; suppress proliferation of MG; promote axon growth; disrupt BRB	*In vitro* human MG study Liu et al. (2015)
		*In vivo* murine studies Xia et al. (2011), Rhee et al. (2013)
IL-4, IL-17 and receptors	Promote inflammation and apoptosis	*In vitro* human MG study Schmalen et al. (2021)
		*In vivo* mice studies Qiu et al. (2016), Qiu et al. (2021)
G-CSF, GM-CSF	To reduce oxidative stress and protect neurons	*In vitro* human MG, RPE, endothelium and ganglion cells studies Kojima et al. (2011), Legacy et al. (2013), Eastlake et al. (2016), Bascuas et al. (2021)
		*In vivo* rats studies Schallenberg et al. (2012), Liu et al. (2020)
Ccl2, Ccl7, Cxcl2, Cxcl10, CX3CL1 and chemokine receptors	To recruit monocytes, neutrophils, T cells; control angiogenesis and fibrosis; induce pro-inflammatory factors	*In vivo* murine and *in vitro* human MG studies Rutar et al. (2011), Rutar et al. (2015), Natoli et al. (2017), Zhang et al. (2018a), Bucher et al. (2020)
TNF-α and TNFR, CD40	To promote inflammation; promote MG reprogram	*In vitro* human MG study Giblin et al. (2021)
		*In vitro* rat MG study Ji et al. (2022)
		*In vivo* mice study Weigelt et al. (2021)
		*In vivo* zebrafish study Lahne and Hyde (2017)
TGF-β and receptors	To induce retinal fibrosis	*In vitro* human MG study Kanda et al. (2019)
		Human retina and *in vitro* human MG Wu et al. (2020a)
		*In vitro* human MG and *in vivo* mice studies Fan et al. (2020)
		*In vivo* zebrafish and mice studies Conedera et al. (2021)
Norrin	To protect neurons; induce neuroprotective growth factors	*In vivo* mice and *in vitro* rat MG study Seitz et al. (2010)
NGF	Promote inflammation	*In vivo* mice and *in vitro* murine MG study Mysona et al. (2013)
GDNF	To protect neurons	*In vivo* murine studies Wu et al. (2002), Gregory-Evans et al. (2009), Touchard et al. (2012), Garcia-Caballero et al. (2018)
MDK, PTN	To promote migration; promote proliferation of MG progenitor cells; regulate retinal development and stress responses	*In vivo* mice and chick study Campbell et al. (2021)
PEDF	To decrease inflammation	*In vitro* rat MG study Zhang et al. (2006)

## MG to the BRB and Extracellular Matrix

MG participate in the integrity of the BRB (Kugler et al., 2021), via different mechanisms including regulating angiogenesis-related factors. There is massive literature about the VEGF, one of the most extensively studied proangiogenic factors, in the MG. There are three evolutionarily related VEGF receptors in humans: VEGFR1 (FLT1), VEGFR2 (KDR/FLK1), and VEGFR3 (FLT4); and five VEGFR ligands (VEGF-A, -B, –C, -D, and placental growth factor (PlGF)) (Uemura et al., 2021). VEGF can be induced by many kinds of stimulations, such as reactive oxygen species (ROS) generated by inflammatory (Li et al., 2019) or with LPS stimulation (Llorian-Salvador et al., 2020). Disruption of VEGF signaling in the MG can introduce complicated consequences. The disruption of VEGFA expression in MG reduces retinal inflammation, vascular lesions, and vascular leakage in diabetic mice (Wang et al., 2010), while the loss of VEGFR2-mediated signaling in MG causes a significant elevation of apoptotic MG, a more severely impaired retinal structure and functions (Fu et al., 2015).

Like VEGF, FGF2 is also a pro-angiogenic factor secreted by MG that promote neovascularization in the retina and choroid, which is generally a sign of disease progression (Campochiaro, 2000; Oladipupo et al., 2014). TNF-α, IL-6 and IL-8 are proangiogenic factors that MG release (Li et al., 2019; Liu et al., 2021). MG also secrete antiangiogenic factors, including PEDF, thrombospondin-1 (TSP-1), tissue inhibitors of metalloproteinases (TIMPs), plasminogenkringle 5, and endostatin, to balance the angiogenesis (Li et al., 2019). MG dysfunction disrupts the balance between the pro- and anti-angiogenic factors, which induces new blood vessels without proper BRB and facilitates retinal inflammation (Li et al., 2019). ICAM-1 (or CD54) is a cell surface glycoprotein and an adhesion receptor which is highly inducible and regulates leukocyte recruitment and adhesion to the vessel from circulation to sites of inflammation (Bui et al., 2020). ICAM-1 has a soluble form, which has been found in the vitreous fluid and associated with intraocular inflammation like DR (Noma et al., 2014). ICAM-1 is a pro-inflammatory factor regulated by NFκB, which was reported to be upregulated in retina with diabetic retinopathy and used for evaluation of the diabetic changes in the retina (Meleth et al., 2005; Shelton et al., 2007). MG was reported to express ICAM-1, which can be upregulated in response to CD40, a member of the TNF superfamily that activates NFκB signaling pathway, indicating its role as a mediator in retinal inflammatory response (Portillo et al., 2014). The kallikrein-kinin system (KKS) plays important roles in neovascularization and retinal inflammation (Othman et al., 2021). Activated MG express kinin B_1_ receptor, which can be induced by activation of NF-κB and the subsequent release of pro-inflammatory cytokines and is detrimental to the retina (Couture et al., 2014; Hachana et al., 2018).

Regulations of ECM is part of the retinal inflammation (Fuchshofer et al., 2005; Fan et al., 2020). The contributions of MG to the ECM also facilitate retinal fibrosis (Roy et al., 2016), which is not a favourable response. The tissue inhibitors of metalloproteinases (TIMPs) and matrix metalloproteinases (MMPs) are the major regulators for ECM. The TIMPs regulate ECM remodelling by controlling the activity of MMPs. TIMPs also act as signalling molecules with cytokine-like activities, influencing cell growth, apoptosis, differentiation, angiogenesis, and oncogenesis (Ries, 2014; Abu El-Asrar et al., 2021b). MG are a source of MMPs, which impair the barrier function of retinal endothelial cells and regulate phospho-ERK1/2, NF-κB, VEGF in MG (Reichenbach et al., 2007). MG also express TIMP-3, which ameliorates high-glucose-induced inflammatory responses by attenuating phospho-ERK1/2, caspase-3, the ADAM17 (a disintegrin and metalloproteinase, also a TNF-α converting enzyme), and VEGF upregulation, and attenuating CCL2 upregulation induced by CoCl_2_ (the hypoxia mimetic agent) and TNF-α (Abu El-Asrar et al., 2021a). The ECM regulation is also mediated by signalling pathways including TGFβ families, which are variable between species and mentioned above.

## Immunocyte-Related Functions in MG

MG have some functions that make them immunocytes. MG can recognize antigens by expressing toll-like receptors (TLRs), a major family of pattern recognition receptors (Kumar et al., 2013), indicating their critical role in innate immunology in the retina, which is important as the retina is generally immune privileged. A study reported that loss of TLR4 in endothelial cells but not MG protects the diabetic retina (Seidel et al., 2021). In another study, however, the inhibition of TLR4 coreceptor myeloid differentiation protein 2 (MD2) was shown to reduce the TLR4-mediated inflammatory factor production, potentially protecting the diseased retina (Chen et al., 2021).

MG are also non-professional phagocyte, which have been shown to phagocytose apoptotic cell bodies during development, foreign molecules, cone outer segments and injured photoreceptors (Bejarano-Escobar et al., 2017). Proteomic data support that MG express proteins associated with the formation and maturation of phagosomes (Schmalen et al., 2021). In line with this, MG are found to be atypical APCs, capable of major histocompatibility complex (MHC) class I and MHC class II expression when activated (Lorenz et al., 2021; Schmalen et al., 2021).

MG are part of the complement system in the retina. MG have been found to express c1q, C3 and complement receptors C3aR and C5aR (Astafurov et al., 2014; Kim et al., 2021). They are also the major contributor of complement activators transcripts, including c1s, c3 and c4, according to a transcriptome study (Pauly et al., 2019). Targeting MG for complement system control could be a potential treatment for AMD, as dysregulation of complement pathways is believed to contribute to AMD (Ambati et al., 2013; Kim et al., 2021).

## MG Metabolism and the Relationship With Retinal Inflammation

Metabolic dysregulation may lead to retinal inflammation (Forrester et al., 2020). MG play a unique role in retinal metabolism. The retina, like many cancers, produces energy from glycolysis even in the presence of oxygen, generating lactate from glucose (Ng et al., 2015). It is interesting that the lactate, despite a metabolic substrate, acts on the G protein-coupled receptor 81 (GPR81, also known as hydroxycarboxylic acid receptor (HCA1 or HCAR1)), which expresses in MG and ganglion cells and responds to inflammatory stimuli (Feingold et al., 2011), may be an important target for retinal inflammation treatment (Kolko et al., 2016).

MG are the main site for neurotransmitter recycling. Extracellular glutamate, the major excitatory neurotransmitter in the retina, is transported to MG and converted to glutamine by the enzyme glutamine synthetase (GS), thus the retina is free from excessive neuron excitation (Reichenbach and Bringmann, 2013). The disturbance of glutamate/glutamine homeostasis in the MG can be introduced by IL-1β, leading to glutamate-induced rod photoreceptor cell death. (Charles-Messance et al., 2020). Reduced retinal GS activity even alters retinal ATP levels together with cytokines (IL-1β and TNF-α) (Shivashankar et al., 2021). MG are also the primary site for serine production, which is the precursor of the inhibitory neurotransmitter glycine and participates in the oxidative stress responses (Zhang T. et al., 2018). Arginase I upregulation in MG increases polyamine production that activates NMDA receptors and induces neuron excitotoxic death (Pernet et al., 2007), which promotes retinal inflammation progress.

The metabolism of retinal lipids, including sphingolipids, is closely related to retinal inflammation (Jun et al., 2019; Simon et al., 2019; Forrester et al., 2020; Simon et al., 2021). Inflammatory responses of MG are regulated by many lipids. To name a few, Resolvin D1 (RvD1), a lipid mediator, induces MG hypertrophy (Trotta et al., 2021), which can activate adenosine receptors. Cultured human MG were shown to be stimulated by linoleic and oleic acid, producing increased VEGF. Linoleic acid also significantly increased IL-6 and IL-8 expression (Capozzi et al., 2016). Co-treatment of palmitic acid with high glucose to the cultured MG potentiated the expression of several DR-relevant angiogenic and inflammatory targets, including PTGS2 (COX-2) and CXCL8 (IL-8) (Capozzi et al., 2018). The inflammatory responses of MG can be inhibited by the cytochrome P450 epoxygenase (CYP)-derived epoxygenated fatty acids (Ontko et al., 2021). Palmitic acid (PA) activation of peroxisome proliferator-activated receptor-β/δ (PPARβ/δ) in human MG *in vivo* leads to the production of pro-angiogenic and/or inflammatory cytokines (Capozzi et al., 2020). Sphingosine-1-phosphate (S1P) is a crucial signal for migration of MG (Simon et al., 2015), which not only participates in the inflammatory responses but is also crucial for MG-dependent retinal regeneration.

Purine is more than a metabolic substrate but also an important mediator for signalling. Purine signalling in MG is mediated by various subtypes of adenosine receptors and nucleotide receptors that are metabotropic or ionotropic. MG release ATP upon activation of metabotropic glutamate receptors and/or osmotic membrane stretching (Wurm et al., 2011). The osmotic mechanism is abrogated under conditions associated with ischemia-hypoxia and inflammation, resulting in swelling and K+ channels alteration of the MG when the extracellular milieu is hypoosmotic, which contributes to macular oedema (Pannicke et al., 2005; Wurm et al., 2011). CD40, a member of the TNF superfamily, can function through the ATP-P2X7 receptor pathway (Portillo et al., 2017). The upregulation of CD40 also induces the release of ATP in MG and triggers in microglia/macrophages purinergic receptor-dependent inflammatory responses that drive the development of retinopathy (Subauste, 2019). Another type of adenosine receptor, Adenosine A_2A_ receptor (A_2A_R), is also expressed by the MG. The inhibition of A_2A_R decreases GFAP upregulation and IL-1β expression of MG, and suppresses ROS overproduction, resulting in attenuation of photoreceptor apoptosis after RD (Gao et al., 2020).

MG highly express proteins involved in retinal iron management, including ceruloplasmin (a multicopper oxidase with ferroxidase activity, facilitating iron export), hephestin (a membrane-bound protein that mediates the uptake of iron from the intestine into circulation), ferroportin and its ligand hepcidin (to export ferrous iron out of cells and convert it to ferric iron) (He et al., 2007; Garcia-Castineiras, 2010). Iron and inflammation are inexorably linked, with ferritin even believed as a pro-inflammatory cytokine (Kernan and Carcillo, 2017). Iron is among the components of drusen in AMD (Ashok et al., 2020). Recently iron overload was found to accelerate the progression of DR (Chaudhary et al., 2018).

## MicroRNA Regulation and the MG

MicroRNAs (miRNAs) are endogenous approximately 22 nucleotides RNAs that can play important regulatory roles by targeting messenger RNAs (mRNAs) for cleavage or translational repression (Bartel, 2004). How miRNAs in adipose tissue regulate chronic inflammation in obese individuals are already discussed (Arner and Kulyte, 2015). In the eyes, miRNAs not only actively participate in the cell fate determination in the retinal development (Cremisi, 2013), but also regulate the differentiation potential of the MG (Quintero and Lamas, 2018). Besides, miRNAs participate in the pathogenesis of multiple diseases, including Leber hereditary optic neuropathy (LHON), autosomal dominant optic atrophy (ADOA), glaucoma, DR, and AMD (Zuzic et al., 2019; Carrella et al., 2021). The miRNA expression profile of mouse MG *in vivo* reveals that miR-204, miR-9, and miR-125-5p are the ones highly expressed by MG but low in neurons (Wohl and Reh, 2016). But they are not the most sensitive ones in regarding inflammation. MiR-124, normally localizing to both INL and OLM of the retina, redistributes from neurons to MG with increased expression under photo-oxidative damage, targeting Ccl2 for retinal inflammation regulation (Chu-Tan et al., 2018; Kang et al., 2020), suggesting its role behind the retinal inflammatory responses. The inflammation-related genes *Fgf2, Mt2* and *Ccl2* are regulated by MG miRNAs and predominantly by members of the let-7 family. Besides, miR-125b-5p, let-7b, and let-7c can be the targets to attenuate gliosis (Kang et al., 2020). miR-320 is another interesting candidate for the regulation of MG inflammatory responses. Up-regulation of miR-320 abrogates HG-induced pro-inflammatory response in MG, while miR-320 inhibitor elevates inflammation in MG cells under hyperglycaemic status (Fu et al., 2021).

Table 2 concluded some typical MG-related microRNAs and how they regulate the retinal inflammation.

**TABLE 2:** MG-derived miRNAs and retinal inflammation.

miRNAs from MG	Roles in the regulation of retinal inflammation	references
miR-124	Correlated with progressive retinal damage, inflammation, and cell death; inversely correlated to Ccl2	Chu-Tan et al. (2018)
miR-125	Regulate the gene *Ccl2*	Kang et al. (2020)
miR-204	To mediate pro-inflammatory cytokine production *via* the miRNA-204/Sirt1 axis	Tu et al. (2020)
miR-320	To abrogate HG-induced pro-inflammatory response in MG	Fu et al. (2021)
Let-7 family	To regulate inflammation-related genes, including *Fgf2*, *Mt2* and *Ccl2*	Kang et al. (2020)

## Treatment for Retinal Inflammation Through MG

Choices of treatments for retinal diseases are expanding over the years, such as intraocular anti-VEGF and corticosteroid administration, which benefit the patients in many cases. But for those that not response to anti-VEFG treatment or already suffer from gliotic scars, more effective options are still needed.

### Retinal Regeneration

MG are not only a regulator of retinal inflammation, which can cause retinal gliosis, but also a potential source of retinal regeneration. MG in zebrafish are capable of regenerating all major retinal neuron types (Wan and Goldman, 2016), while in mammals the potential of retinal regeneration from MG is limited (Jorstad et al., 2017; Yao et al., 2018). Factors affecting MG reprogramming and proliferation are reviewed elsewhere (Goldman, 2014), some of which are also inflammation-related, such as CNTF, FGF2 and TGF-β. It would be of great significance if we can manipulate the responses of the MG to drive retinal regeneration rather than gliosis, which can be detrimental to the retina. The MG-dependent regeneration was controlled by acute inflammation, microglia and immune system (Silva et al., 2020; Todd et al., 2020), involving the inflammation-related signaling pathways, including NF-κB signaling and mTOR signaling (Palazzo et al., 2020; Zhang et al., 2020). Some of the signaling pathways are regulated in different ways in different species and may be the key for retinal regeneration induction. For example, upon photoreceptor injury in the zebrafish retina, mmp-9 is induced in MG and MG-derived photoreceptor progenitors (MGDP) (Silva et al., 2020). In mammals, however, MMP-9 functions as a crucial ECM regulator that promotes angiogenesis in DR (Kowluru et al., 2012; Kowluru and Mishra, 2017), a suppressor of the anti-angiogenetic factor opticin (Le Goff et al., 2012; Patnaik et al., 2021), inducing laminin degradation and neuron apoptosis (Wu L. et al., 2020), and keeping BRB integrity (Chen et al., 2008). Similarly, among the TGF-β superfamily, TGF-β3 in MG is upregulated after retinal injury and promotes retinal in zebrafish, while in mice, TGF-β1 and TGF-β2 are activated in MG and evoke the p38MAPK signaling pathway during gliotic responses (Conedera et al., 2021). The causes of the different patterns of responses among different species remain largely unknown. Understanding the mechanisms that cause the differences may help to find ways of promoting retinal regeneration instead of gliosis after acute inflammation, which would be a potential treatment for some retinal diseases involving gliosis. Methods of treatment that targets only MG without systemic side effects are also worth exploring. MG or MG-derived retinal neurons have led to improvement of retinal function after experimental transplantation (Eastlake et al., 2021). The neuroprotective effects observed are mostly attributed to the neurotrophic factors derived from the transplanted cells and lasts for limited period of time. The induction of true regeneration in mammals requires deeper understanding of cellular processes including migration and integration of grated cells to the hosts (Eastlake et al., 2021). Despite the difficulties, MG-dependent retinal regeneration is still a promising future direction for degenerative retinal diseases, whether they are inflammation-related or not.

### Novel Treatments Targeting MG Inflammatory Responses

Corticosteroids are the classical treatment for retinal inflammation. Fludrocortisone (FA) and triamcinolone acetonide (TA) reduce the expression of CCL2, IL-6, and IL-8 in MG *in vitro* after inflammatory challenge with IL-1β or TNF-α, suppressing inflammation and potentially neurodegeneration (Racic et al., 2021). More targets are being explored for MG-released cytokines reduction. The nuclear factor of activated T-cells (NFAT) is a transcriptional regulator of inflammatory cytokines and adhesion molecules. Targeting NFAT attenuates IL-1β-induced pro-inflammatory cytokines secretion from the MG (Giblin et al., 2021). HspB4/αA-crystallin, a molecular chaperone highly expressed in glial cells in the retina, regulates the stress-induced expression of pro-inflammatory cytokines in MG through the modulation of multiple key inflammatory pathways, suggesting its potential as a therapeutic target for the modulation of chronic neuroinflammation (Nath et al., 2021).

Some classical drugs are proved to be beneficial for retinal inflammation control. For example, the traditional Chinese medicine, Mingmu Xiaomeng tablets reduce serum levels of interleukin (IL)-1β, IL-4, IL-6, TNF-α, and VEGF (Fang et al., 2021). Also, the vitamin thiamine confirms once more to be an effective agent in reducing diabetes-induced retinal damage (Mazzeo et al., 2020). CXCL1 upregulation in MG, as well as endothelial, can be impaired by a peptide blocking the CD40-TNF receptor-associated factors (TRAF) 2,3 signaling (Portillo et al., 2021), which can potentially be used for inflammation control. Some interesting studies show novel treatments targeting MG for retinal inflammation control. Photobiomodulation with 670 nm light was shown to ameliorate MG-mediated activation of microglia and macrophages in retinal degeneration (Lu et al., 2017). The adipose-derived stem cell (ASC) concentrated conditioned medium (ASC-CCM), containing various components released from stem cells, normalizes GFAP, viability, and catalase activity oxidatively stressed MG, indicating its potential for retinal inflammation control (Jha et al., 2022). The pan-retinoid X receptors (RXR) agonist PA024 reduces MG reactivity, decreases GFAP and increases GS expression, promoted rd1 photoreceptor survival in retina neuro-glial cultures (Volonte et al., 2021). Other targets including miR-192 and Nurr1 (Li et al., 2020; Gu et al., 2021).

## Discussion

**FIGURE 1:**
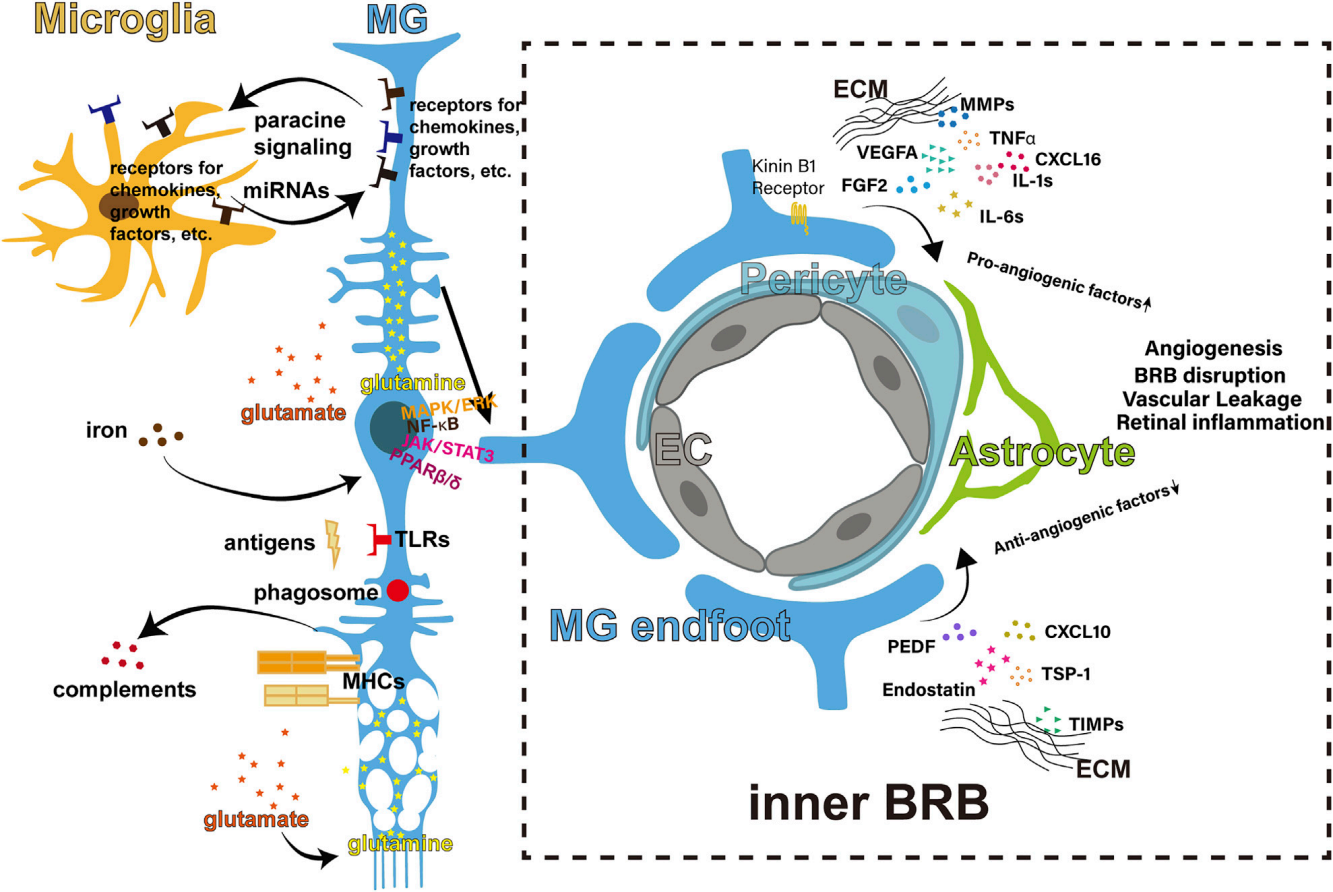
Regulations of retinal inflammation from the MG. The body of the MG spans across the retina. The MG interact with the microglia in the inner retina through cytokines paracrine signaling and miRNAs. The main signaling pathways involved include MAPK/ERK, NF-κB, JAK/STAT3 and PPARβ/δ. The MG intake the glutamate released by neurons and convert the neurotoxic glutamate to non-toxic glutamine. The MG take in excessive iron and prevent iron-induced inflammation. The MG express TLRs and recognize antigens after infection, and are capable of phagosome-dependent clearance. The MG can express MHCs and release complements. The endfoot of the MG is crucial part of the inner BRB, regulating angiostasis through pro- and anti-angiogenic factors. BRB, blood-retinal barrier; EC, endothelial cells; EMC, extracellular matrix; MHC, major histocompatibility complex; MG, Müller glia; miRNA, micro-RNA.

Retinal inflammation is involved in the pathogenesis of many retinal diseases. Figure 1 concluded the mechanisms discussed above. MG participate throughout the processes of retinal inflammatory responses. MG sense chemical (cytokines, etc.), physical (osmolarity, etc.), and biological (antigens, etc.) stresses by their receptors. MG react to the stresses by secreting cytokines and inflammation-related factors, regulating retinal ECM and BRB, modulating their metabolism, all of which contribute to different directions of retinal inflammatory responses. The responses of MG are controlled by sophisticated mechanisms including the inflammatory pathways and miRNAs.

Targeting MG for retinal inflammation treatment is worth more investigation. Most of our studies have focused on the deleterious effects of the release of pro-inflammatory factors on the retina with the aim of removing inflammatory mediators. The protective properties of MG-derived growth factors and cytokines, such as CNTF, GDNF and LIF, can be utilized to protect the neural retina if they can be properly controlled, especially for diseases that involve chronic inflammation, such as AMD. Besides, the mechanisms of how the MG microenvironment contributes to the ECM remain largely unknown. Knowing the contributions of MG to the ECM not only help to regulate the ECM-related inflammatory pathways, but also benefit the BRB integrity maintenance. The mechanisms that lead to different reactions of MG to injury or inflammation between different species are also worth exploring, as the switch from the gliosis to regeneration can be a promising treatment for degenerative retinal diseases like AMD and DR. Except for the TGF pathways mentioned above, some of the inflammation-related miRNAs (such as let 7 families, miR-124) in MG have been found to contribute to MG-dependent retinal regeneration (Wohl et al., 2019). It would be interesting to investigate the difference between the profiles of MG-derived miRNAs induced by injury or acute inflammation among different species. The delivery of exogenous factors to promote retinal regeneration can be subtle but long-lasting methods are needed. Treatments by manipulating the responses of endogenous MG avoid side effects brought by transplantation, such as immune rejection, but the off-target effects must be carefully monitored, as the changes in MG may disturb the integrity of BRB and lead to systemic reactions.
